# Macromolecular Crowding Is Surprisingly Unable to Deform the Structure of a Model Biomolecular Condensate

**DOI:** 10.3390/biology12020181

**Published:** 2023-01-25

**Authors:** Julian C. Shillcock, David B. Thomas, John H. Ipsen, Andrew D. Brown

**Affiliations:** 1 Blue Brain Project and Laboratory of Molecular and Chemical Biology of Neurodegeneration, Ecole Polytechnique Fédérale de Lausanne, CH-1015 Lausanne, Switzerland; 2 Department of Electronics and Computer Science, University of Southampton, Highfield, Southampton SO17 1BJ, UK; 3 Department of Physics, Chemistry and Pharmacy, University of Southern Denmark, Campusvej 55, DK-5230 Odense M, Denmark

**Keywords:** liquid–liquid phase separation, biomolecular condensate, crowding, coarse-grained simulation, event-based computing, hardware-accelerated simulation

## Abstract

**Simple Summary:**

The cytoplasm of a living cell is a crowded place, containing hundreds of types of protein and other macromolecules. Cells reliably and continually perform thousands of biochemical reactions to maintain their health. Biomolecular condensates are fluid protein compartments that provide distinct local environments, within which they carry out cellular functions. How they prevent their contents mixing with the external environment without being encapsulated inside a lipid membrane is not fully understood. Many researchers approach this question by studying simpler systems in a test tube that contain only a few protein types although it is hard to relate their results to the complex cellular milieu. Computer simulations are used to explore the predictions of simple models of cellular behavior, but are also limited by the ability of human experimenters to recreate important aspects of the cytoplasm, in particular, its crowded nature. We have used a novel computer framework to perform dozens of simultaneous simulations that map out the influence of macromolecular crowding on the formation and structure of a biomolecular condensate. We find that the spatial structure of the model condensate is surprisingly insensitive to the composition and concentration of external macromolecules, even when its formation is assisted by steric repulsion from its environment.

**Abstract:**

The crowded interior of a living cell makes performing experiments on simpler in vitro systems attractive. Although these reveal interesting phenomena, their biological relevance can be questionable. A topical example is the phase separation of intrinsically disordered proteins into biomolecular condensates, which is proposed to underlie the membrane-less compartmentalization of many cellular functions. How a cell reliably controls biochemical reactions in compartments open to the compositionally-varying cytoplasm is an important question for understanding cellular homeostasis. Computer simulations are often used to study the phase behavior of model biomolecular condensates, but the number of relevant parameters increases as the number of protein components increases. It is unfeasible to exhaustively simulate such models for all parameter combinations, although interesting phenomena are almost certainly hidden in their high-dimensional parameter space. Here, we have studied the phase behavior of a model biomolecular condensate in the presence of a polymeric crowding agent. We used a novel compute framework to execute dozens of simultaneous simulations spanning the protein/crowder concentration space. We then combined the results into a graphical representation for human interpretation, which provided an efficient way to search the model’s high-dimensional parameter space. We found that steric repulsion from the crowder drives a near-critical system across the phase boundary, but the molecular arrangement within the resulting biomolecular condensate is rather insensitive to the crowder concentration and molecular weight. We propose that a cell may use the local cytoplasmic concentration to assist the formation of biomolecular condensates, while relying on the dense phase to reliably provide a stable, structured, fluid milieu for cellular biochemistry despite being open to its changing environment.

## 1. Introduction

How the myriad biochemical reactions that support cellular life are spatially organized is a fundamental question in cell biology. It is equally crucial for a cell to regulate reactions despite the crowded nature of the cytoplasm. Estimates of the concentration of proteins in eukaryotic cytoplasm range from 100–450 mg/mL, which implies that specific macromolecular interactions compete with many random processes involving other molecules [1,2,3]. Confining reactions inside discrete compartments is one solution to these problems. The nucleus, endoplasmic reticulum, mitochondria, and many other organelles. are wrapped in a phospholipid membrane that segregates their specific biochemistry. In the last decade, an older idea of cellular compartmentalization has re-emerged as a paradigm for cytoplasmic organization [4,5,6,7,8]. Dense protein droplets composed of distinct mixtures of proteins (and often RNA or DNA) have been found to carry out many cellular functions [9,10,11,12]. The proteins that form them lack a minimum-energy folded state, but instead sample a wide ensemble of similar-energy conformations in dilute solution [13,14]. This has led to their being labelled intrinsically disordered proteins (IDPs). Unlike the organelles mentioned above, these droplets, which are commonly referred to as biomolecular condensates (BCs), have no bounding phospholipid membrane to prevent molecular mixing with the cytoplasm. It is recognized that BCs do not completely isolate their interior from the cytoplasm. Recent work has shown that some kinases respond to molecular crowding in the cytoplasm by condensing into a functional BC that facilitates a signaling pathway to regulate the cell volume [15]. Crowding is also known to influence protein folding [16]. Aberrant phase transitions of IDPs are implicated in neurodegenerative diseases, cancer, prion protein diseases, and more speculatively, in ageing [17,18,19,20,21]. Experiments on BCs have advanced to the point that they are being synthetically designed to modulate biochemical and cellular functions [22,23,24,25,26,27,28] and mined for novel drug targets [29,30,31]. A better understanding of how BCs are coupled (or not) to their crowded environment would assist in deciphering their cellular roles and constructing synthetic BCs [32,33,34,35,36,37,38].

The complexity of living cells has driven research on model condensates, which typically use unphysiologically high concentrations of one, or at most a few, species of IDP in a buffer, although their relevance to living cells has been strongly questioned [39,40]. An example is provided by in vitro experiments on the protein fused in sarcoma (FUS), an RNA-binding protein that is implicated in the neurodegenerative disease ALS [41,42,43,44]. Computational modelling has also been used to study the phase behavior of FUS [43,45,46,47,48,49,50]. However, models that are sufficiently complex to be biologically relevant suffer from a common limitation: their parameter space is huge because many properties of the constituent molecules might a priori be important for their behavior [51]. A popular conceptual model for IDPs is the so-called stickers and spacers model, in which the molecules are regarded as semi-flexible polymers with multiple attractive domains (stickers) that are connected by weakly interacting linkers (spacers) [9,45,46,52,53]. When such models aim for one-monomer-per-residue accuracy, they require specifying ~400 interaction parameters just to describe the pairwise forces between the 20 amino acid types [46].

Further coarse-graining the stickers and spacers model reduces the force field complexity, but still requires assigning values to multiple parameters, including the IDP molecular weight, concentration, backbone stiffness, location of the multiple binding sites, and the interaction energies between all species (solvent, backbone bead, and binding sites). We have previously used Dissipative Particle Dynamics (DPD) simulations to explore the phase behavior and structure of a model biomolecular condensate formed of a single type of IDP modelled on the FUS low-complexity domain (FUS LC) [50,54,55,56]. Here, we ask the simplest question related to the response of a biomolecular condensate to its environment: how does its phase behavior and internal structure respond to the crowding effect of other macromolecules? 

Naively adding additional molecule types as crowding agents multiples the parameters geometrically, rendering it impossible to explore the parameter space of the model in a systematic way. We overcome this barrier by performing multiple, parallel simulations on a novel compute framework [57,58]. Our goal is to rapidly locate interesting regions of the parameter space and make experimentally relevant predictions, while minimizing the computational cost and experimentation time. The simulated FUS-LC molecules are based on previous work [50], and a soluble polymeric crowding agent (referred to hereafter as the *crowder*) is added, which exerts a steric pressure on the IDP molecules (and itself), but is otherwise inert.

Even after these simplifications, the system has a six-dimensional parameter space—two molecular weights, two concentrations, the IDP self-attraction, and the crowder/IDP repulsion strength— which may affect the phase behavior in a non-trivial way. For simplicity, we set the IDP and crowder molecular weights to be equal and initially fix the crowder/IDP repulsion to a high value (see Table 1). This leaves three parameters to be explored in the simulations: the crowder and IDP concentrations and the IDP self-attraction.

A final difficulty, which is intrinsic to all computational models with many parameters, is our inability to simultaneously visualize multiple states of the model as the parameters vary. We show that by using parallel hardware to accelerate dozens of simulations and displaying the results in a grid of visually rich simulation snapshots, we can rapidly locate the fruitful regions of the parameter space. Our results predict that the spatial arrangement, but not necessarily the dynamics, of the IDPs within the model condensate is surprisingly insensitive to the crowdedness of its environment, even when steric repulsion from the crowder is required for its formation. Biomolecular condensates, therefore, provide a stable, structured fluid environment for biochemical reactions because the molecular structure of IDPs decouples their internal state (to a certain degree) from their compositionally-varying surroundings.

## 2. Materials and Methods

### 2.1. Dissipative Particle Dynamics Simulations

The Dissipative Particle Dynamics simulation technique (DPD) was invented to study complex fluids such as polymer mixtures, phospholipid membranes, diblock copolymers, etc. [54,55,59,60,61]. The simulation source code we use is available on Github [62]. DPD is a coarse-grained molecular technique in which atoms are aggregated into beads that interact via three effective forces. All of the beads have mass m, and the three non-bonded forces between them are soft, short-ranged (vanish beyond a fixed length-scale $d_{0}$), pairwise additive, and conserve linear momentum. A conservative force gives each bead an identity:

(1) $$F_{ij}^{C}=a_{ij}\left(1-r_{ij}/d_{0}\right)\;{\hat{r}}_{ij},$$

for $r_{ij}<d_{0}$. In this equation, $a_{ij}$ is the maximum value of the force; $r_{ij}=r_{i}-r_{j}$ is the relative position vector from bead *j* to bead *i*, $r_{ij}$ is its magnitude, and ${\hat{r}}_{ij}$ is the unit vector directed from bead j to bead i. Two other non-bonded forces constitute a thermostat that ensures the equilibrium states of the simulation are Boltzmann distributed. The dissipative force is:

(2) $$F_{ij}^{D}=-\gamma_{ij}\;{\left(1-r_{ij}/d_{0}\right)}^{2}\left({\hat{r}}_{ij\;}.\;v_{ij}\right).\;{\hat{r}}_{ij\;},$$

where $\gamma_{ij}=4.5$ is the strength of the dissipative force, which is the same for all of the bead types, and $v_{ij\;}$ is the relative velocity between beads i and j. The random force is:

(3) $$F_{ij}^{R}=\sqrt{2\gamma_{ij}k_{B}T/dt}\;\left(1-r_{ij}/d_{0}\right)\;\zeta_{ij}\;{\hat{r}}_{ij},$$

where $k_{B}T$ is the system temperature and $\zeta_{ij}\;$ is a symmetric, uniform, unit random variable that is sampled for each pair of interacting beads and satisfies $\zeta_{ij}=\zeta_{ji}$, $\zeta_{ij}\left(t\right)=0$, and $\zeta_{ij}\left(t\right)\zeta_{kl}\left(t’\right)=\left(\delta_{ik}\delta_{jl}+\delta_{il}\delta_{jk}\right)\delta \left(t-t’\right)$. The factor $1/\sqrt{dt}$ in the random force ensures that the discretized form of the Langevin equation is well defined. 

Once the required bead types have been specified, they are connected into molecules by tying them together with Hookean springs, whose energy function is:

(4) $$U_{2}\left(i,\;i+1\right)=\left(1/2\right)k_{2}{\left(r_{i\;i+1}-l_{0}\right)}^{2},$$

and the spring constant,$\;k_{2}$, and unstretched length,$\;l_{0}$ are fixed at the values $k_{2}=128\;k_{B}T/d_{0}^{2}$ and $l_{0}=0.5\;d_{0}$ for all of the bond types. Finally, because the peptide chains that form the backbone of the IDPs have a bending stiffness, we add a chain bending potential to the angle φ, which is defined by adjacent backbone bead triples (BBB) with the energy function:

(5) $$U_{3}\left(i-1,\;i,\;i+1\right)=k_{3}\left(1-cos\left(\varphi -\varphi_{0}\right)\right),$$

with the parameters $k_{3}=5$ $k_{3}=5$ and $\varphi_{0}=0$. 

**Figure 1:**
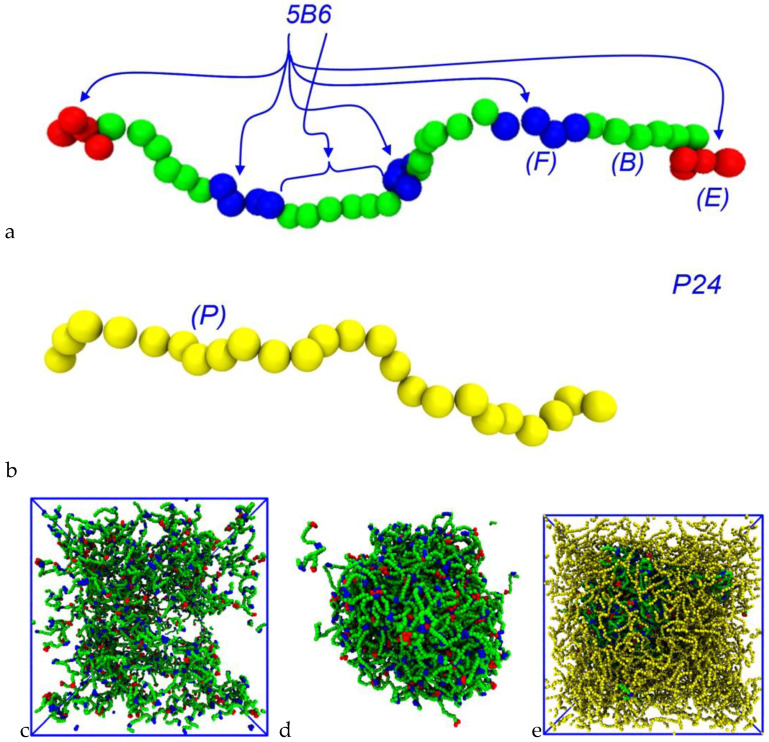
Molecular structure of (**a**) 5B6 IDP and (**b**) P24 crowder molecule. The notation 5B6 indicates that there are five binding sites, and adjacent ones are separated by six backbone beads. The endcaps and internal binding sites are colored differently for clarity. The crowder polymer beads are yellow and strongly repulsive towards themselves and all of the IDP bead types (see Table 1). All of the IDP and crowder bead types are hydrophilic. (**c**) Snapshot of a dispersed system containing 250 IDPs of type 6B10 with no crowder and (**d**) 234 IDPs of the same type in the presence of 468 P48 crowder molecules. (**e**) Similar IDP/crowder system with visible crowder polymers. Note, the simulation box is not shown, and crowder molecules are invisible in panel (**d**). Solvent particles are invisible in all figures for clarity.

Three types of molecule are defined in the simulations. The IDPs are semi-flexible, linear polymers containing a set of binding sites (*sticky* bead types E for the endcaps and F for the internal binding sites), which are separated by segments of inert backbone beads (B). The crowding agent is a self-avoiding, semi-flexible homopolymer composed of a single bead type (P). The solvent molecules are represented by a single bead (W). Both the sticky beads and the backbone beads of the IDP and crowder beads are hydrophilic, and we emphasize that there is no hydrophobic repulsion of the IDPs or crowder from the solvent. In the notation of [50], 5B6 represents an IDP containing 5 binding sites (including the endcaps) that are separated by 6 backbone beads (Figure 1), and similarly, 6B10 is an IDP with 6 binding sites that are separated by 10 backbone beads. The crowder polymers diffuse freely throughout the simulation box, exerting an osmotic pressure on the IDPs. The crowder molecules composed of 12, 24, and 48 monomers are indicated by the notations P12, P24, and P48, respectively. All of the non-bonded conservative interaction parameters are given in Table 1. As in the previous work [50], we quantify the attraction of the IDP binding sites by defining a dimensionless parameter ϵ in terms of the conservative force parameter for the binding sites’ self-interaction and interaction with the solvent, namely, $\epsilon =\left(a_{EW}-a_{EE}\right)/a_{EW}$. A value of ϵ=0 means there is no net attraction between the sticky sites, as they have the same interaction with each other as they do with the solvent beads. Higher values of ϵ  indicate a stronger attraction. We point out here that bead types E and F have identical interactions in the simulations, but are labelled differently for visual clarity in exploring the phase behavior.

Simulations take place in a cubical box with dimensions 48 × 48 × 48 (d_0_)^3^, unless otherwise stated, and periodic boundary conditions are applied. The phase behavior of the model IDP/crowder system is studied by distributing a given number of IDPs and crowders randomly throughout the simulation box and filling the remaining space with solvent particles to an average density of $\rho d_{0}^{3}=3.\;$ All beads have mass *m* = 1, and the reduced system temperature is $k_{B}T=1$. Each simulation is performed for one million time steps using an integration step size of 0.02 τ, where $\tau =\sqrt{md_{0}^{2}/k_{B}T}$ is the DPD timescale. The first half of all of the simulations is discarded and equilibrium averages are constructed from samples taken from the second half. Some simulations are extended to two million steps or longer to ensure they reach equilibrium.

### 2.2. POETS 

Exploring multiple parameter models is an iterative process involving a human–computer interaction, with the computer performing simulations on a chosen set of parameter values, followed by the human evaluating the results and choosing new sets of parameter values. This type of human-driven parameter space exploration presents both computational and experimental design problems:

1. How do we compute multiple simulations fast enough to support semi-interactive parameter space exploration?
2. What visualization and workflow support can be created to support a human who wishes to perform such an exploration?

We solved the first problem by running multiple instances of a new high-performance DPD simulator in a batch processing system, allowing for the rapid simulation of multiple parameter sets in parallel. The second problem is solved using an automated execution and visualization workflow wrapped around the parallel simulators, minimizing the human effort needed to manage and post-process the results during the search. This approach allowed us to simulate and visualize the results of a 4 × 5 grid of parameters (a 2D parameter space slice) in two hours, while reducing the analysis time needed by the human to just five minutes for the visual inspection. This two hour process should be compared to the 7 days that are needed if the 20 simulations are executed sequentially.

Both the simulator and visualization framework were inspired by a collaborative research project called POETS [58], which is an ongoing project exploring a new computing paradigm called event-triggered computing. In event-triggered computing, the applications and hardware are designed around the frequent exchange of events (small messages) between small asynchronous state machines, rather than the infrequent exchange of large messages between threads (as seen in MPI).

The main idea of POETS is to execute event-triggered applications using custom-built hardware, allowing applications to efficiently execute on thousands of lightweight threads. This idea has previously been applied successfully to DPD simulation, where it was used to tackle simulations of a large spatial size [51]. However, while we were performing that research, we uncovered an opportunity and a challenge: the opportunity was that the event-triggered algorithms were also a surprisingly good fit for modern multi-core CPU architectures. The challenge was that there is currently only one large POETS hardware system, for which there is significant competition for access time.

The fit between the event-triggered algorithms and contemporary multi-core CPUs also resulted in a very fast, multi-core, shared memory DPD simulator. The technical details are not the focus of this paper, but one of the main ideas is that it uses a very fine-grained spatial domain decomposition, so that each thread manages a unit volume cell. As beads move around in the simulation they are also moved between the cells in memory, rather than building and maintaining edge lists. The event-triggered nature of the algorithm makes it particularly amenable to SIMD vectorization, reduces the cache traffic, and allows for efficient shared memory multi-threading.

We do not make claims about the relative efficiency of this approach for all computational problems, but for the experiments that were performed in this work using the Iridis data center (https://www.southampton.ac.uk/isolutions/staff/iridis.page, accessed on 16 January 2023) our approach resulted in a 2× speed-up compared with the industry-standard code LAMMPS (https://www.lammps.org, accessed on 17 January 2023) in a single 64-core system. Two important aspects of this setup were:

- It allowed the experiments to be completed in under two hours, which allowed them to be submitted to the “fast” low-latency queue in the Iridis job manager;
- It means we do not need to use GPUs to achieve low latency, which is useful because GPUs are less common in many HPC clusters and they are heavily subscribed by chemists, physicists, and machine learning researchers. In our experience, it typically takes more than 2 hours for a GPU job to even start running, even though it may perform faster once it has started.

So, the result of having a very fast, multi-core, shared memory is that multiple jobs can be issued and completed very quickly. Clearly, these exact figures are not true of all HPC systems, but it does address two common problems: GPUs are a very contested resource, and the jobs need to have short run times in order to achieve low latency.

This multi-core oriented approach allows 20 independent 48 × 48 × 48 × 1 M simulations to be completed in under 2 hours, which is almost interactive; in principle a researcher could perform four simulate–observe–decide iterations during a working day. In practice, we found that there was substantial overhead due to preparing the simulation inputs, collecting the outputs, and then post-processing them into a useable form that a human could analyze. Simple tasks such as ordering and organizing images take large amounts of time, involving the researchers opening multiple files and trying to arrange them in some way to support the interpretation and analysis. Creating and submitting the next batch of simulations is then another time consuming step.

**Figure 2:**
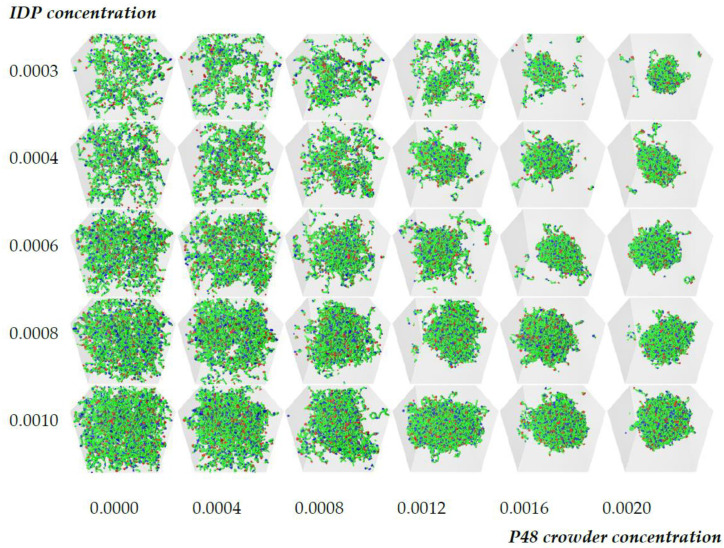
Illustrative grid of snapshots of 6B10 molecules with constant affinity ε=0.6 in the [Crowder]-[IDP] plane. The rows have constant IDP concentrations (top to bottom): 0.0003, 0.0004, 0.0006, 0.0008, 0.001, and the P48 crowder concentration increases across the columns (from left to right): 0.0, 0.0004, 0.0008, 0.0012, 0.0016, and 0.002. Increasing the crowder concentration (top row) drives the system across its phase boundary, producing a dense droplet surrounded by a dilute phase. Examining the lower rows of the grid reveals that the crowder concentration required to drive phase separation decreases with an increasing IDP concentration (bottom row). Solvent and crowder molecules are invisible in all snapshots for clarity.

Through experimentation we found that the most effective approach—at least for our current purposes—is to explore a plane of two parameters, and then automatically arrange the simulation outputs into a visual 2D grid (e.g., see Figure 2). In most cases, this allows the human to immediately explore and interpret the results. If the current parameter ranges are not exposing any behaviors of interest, a new set of parameters can be found and re-submitted for exploration. If an interesting set of parameters has been discovered, then either more detailed simulations can be performed in the same area or the human can take the simulation results and start to explore them using other approaches for the statistical and visual analysis. 

The overall workflow we developed for the results used in this paper is:

1. Manual: Define a model with multiple parameter dimensions to sweep;
2. Manual: Construct a parameterized scenario generator that can instantiate the model for specific parameter values;
3. Perform human–computer collaborative search:
  a. Manual: Identify two interesting parameter dimensions and ranges; pick X points for one parameter and Y points for the other parameter and generate the XxY concrete scenarios to be simulated;
  b. Automatic: Simulate the scenarios in parallel using multiple machines in a HPC system;
  c. Automatic: Collect the outputs and produce tiled XxY images and videos;
  d. Manual: Inspect the tiled images to understand the parameter response. If necessary, repeat step 3.a.
4. Manual: explore and analyze the results in more detail.

The two manual bottleneck tasks in this iterative process are steps 3.a and 3.d: picking the parameter values at the start of each iteration and manually investigating the results, respectively. Using a set of scripts, we simplified step 3.a down to the point where the user submits a zip file of the simulation scenarios. Each simulation scenario in the zip is an Osprey DPD simulation description file, with a file name prefix indicating its position within an integer grid. In step 3.d, the user then receives back a zip file containing periodic snapshots of each simulation state, along-side automatically assembled image grids of the rendered images showing the spatial variations (see Figure 2). In many cases, it is sufficient to simply open and view the image grid to obtain a sense of the parameter dependency and immediately produce the next set of scenarios.

The automated tools supporting steps 3.b and 3.c are implemented using a combination of the POETS DPD software simulator, python scripts to assemble image grids, and a set of shell scripts to manage them. The grids are executed on the Iridis-5 HPC cluster at the University of Southampton, with one 64-core AMD node per simulation. Each node is able to complete a 48 × 48 × 48 × 1 M simulation in less than 2 h, requiring about 3.3 × 10^11^ bead steps. A grid of 4 × 5 simulations typically completes 6.6 × 10^12^ bead steps in 2 hours.

This workflow could usefully be replicated using other simulation tools. For example, it can be performed using Osprey DPD, though it would slow the iteration time down to at most one iteration per day. It could also be implemented using a GPU accelerated simulator such as LAMMPs, and in an HPC system with abundant GPUs this could be a good solution. However, the GPU nodes are still often a small subset of the total number of computer nodes in many HPC pools, so even if the individual simulation tasks are executed more quickly, the tasks may spend a long time waiting for the execution.

To give a concrete example, in the Iridis-5 system, there are 572 multi-core CPU compute nodes, but there are only 20 GPU nodes, which is not an uncommon balance for a large institution-wide HPC pool for batch computing. As a result, there is intense competition for GPU access time due to physicists, chemists, engineers, and machine learning researchers all wanting to run long multi-hour tasks, so the GPU tasks may spend 10–20 h waiting in the submission queue. To complete a grid of 20 GPU tasks, it might take 2–3 days, even though it only takes 20 h of on-node time, thereby eliminating the interactive aspect of parameter space exploration. By using fast multi-core software simulators we can rapidly execute all of the tasks, exploiting the large number of CPU compute nodes available for short-lived tasks, and complete all 20 tasks in a semi-interactive two hour timescale.

## 3. Results

### 3.1. Crowding Assists Phase Separation of IDPs with Sub-Critical Affinity

Phase separation of IDPs is driven by transient attractive interactions between specific amino acids, such as arginine and tyrosine, and non-specific electrostatic and hydrophobic interactions between residues [63]. It is assisted by the relatively small entropic cost when proteins move from a dilute phase into the dense phase, as demonstrated by FUS that retains high conformational flexibility inside its dense phase [42,43]. In previous work, we showed that model IDPs similar to those shown in Figure 1 spontaneously phase separate when the affinity of their binding sites exceeds a threshold that depends on the number and location of the binding sites [50]. When their affinity is reduced below the critical value, no spontaneous phase separation occurs. Here, we first explored how the presence of a crowding agent influences the phase separation of a model IDP whose affinity is below the critical value.

The model IDP has six binding sites that are separated by ten backbone beads and is represented by the notation 6B10. Previous work showed that the critical affinity for such molecules lies in the range ε=0.68−0.74 [50]. We set the binding site affinity to ε=0.6, which is below the critical value, so the IDPs were unable to phase separate spontaneously. The crowding polymer was P48, and all of the other parameters were fixed as described in the Methods section. This left a two-dimensional parameter space, the IDP and crowder concentrations, which can conveniently be displayed as a two-dimensional array of results. Figure 2 shows a grid of snapshots taken from 30 simultaneous simulations of 10^6^ time steps performed using POETS-DPD (see Methods). The rows have constant IDP concentration, which increases towards the bottom, and the columns have constant crowder concentration, which increases to the right. The crowder molecules are invisible in the figure for clarity, but Supplementary Figure S1 shows the complete systems. The top row shows the general trend in the observed equilibrium states as the crowder concentration is increased. In the absence of a crowder, the IDPs are dispersed (top left image), while increasing the crowder concentration eventually drives the system across the phase boundary, resulting in a phase separated droplet surrounded by a dilute phase (top right image). The same trend is seen in the lower rows of the table for higher IDP concentrations. However, when the IDP concentration is sufficiently high, they already form a loose network that spans the box in the absence of the crowder, a result that has previously been reported in the literature [45,50,56]. Visually comparing all of the snapshots in the grid shows that higher concentrations of IDP phase separated at lower crowder concentrations. When the same IDP/crowder concentrations were simulated with higher affinities ε=0.68, 0.76, the boundary between the dispersed and phase separated states shifted to lower crowder concentrations with increasing affinity (cp. Supplementary Figure S2).

It might be expected that a sufficient concentration of the repulsive crowder could drive phase separation of IDPs whose affinity is low or even zero. This would represent a generic polymer phase separation driven by the steric repulsion between the molecules, as described by Flory–Huggins theory [64]. In particular, it would not depend on the affinity and number of IDP binding sites, and therefore, the heterogeneous spatial organization of the molecules in the dense phase observed in earlier work might be eliminated [50]. To test this possibility, we ran control simulations in which the binding affinity of the IDPs was initially set to a value that is known to drive phase separation (ε=0.68), and subsequently removed after 500,000 time steps (ε=0). Supplementary Movie SM1 shows the evolution of the system with [IDP] = 0.0006 (180 molecules) and [Crowder] = 0.0012 (361 molecules), which corresponds to grid element 3, 4 in Figure 2. When the affinity was removed, the dense phase dissolved, showing that the presence of the crowder alone was unable to drive the phase separation for this combination of IDP/crowder molecular structure and concentrations. This does not preclude that a higher crowder concentration could drive phase separation. Supplementary Movie SM2 shows the result for grid element 3, 6 in Figure 2, showing that the higher crowder concentration of 0.002 (583 molecules) was sufficient to keep the IDPs from dispersing in the absence of any self-attraction between the IDPs. However, when we analyzed this dense phase, the binding sites of the IDPs did not form junctions, and the IDPs are not in a connected network. 

We calculated the crowder concentrations as follows. The densest case in the grid, which corresponds to the rightmost column in Figure 2, has between 568 and 594 molecules of the P48 crowder in the simulation box (the precise value depends on the number of IDP molecules). The molecular weight of the crowder was chosen to be equal to that of the IDP, which represents FUS-LC, and its concentration was calculated using the method in [50]. This gave a concentration of 7 mM, which is equivalent to 120 mg/mL, and is in the range of estimates for the eukaryotic cytoplasm of 100–450 mg/mL [1,2].

### 3.2. Quantitative Properties of Condensate Structure Are Insensitive to Crowder Concentration

We next explored whether the internal organization of the IDPs in the dense phase is modified when the phase separation is assisted by the crowding agent compared to when it forms spontaneously (i.e., when the IDPs have a higher affinity). We previously found that the dense phase has a heterogeneous structure in which the binding sites of the IDPs meet at spatially-discrete junctions whose separation depends on the spacing of the binding sites but is less sensitive to their affinity (cp Figure 3 in [50]). Further, the average number of IDPs that span two junctions increases with increasing binding site affinity and decreasing separation. These junctions are transient because the IDPs continually fluctuate and diffuse through the dense phase.

**Figure 3:**
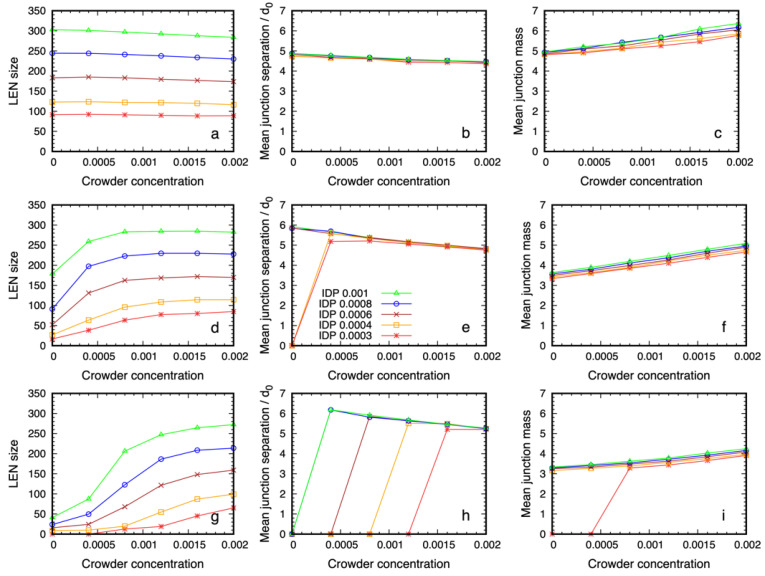
Structural data on the dense phase of 6B10 IDPs with affinities ε=0.6, 0.68, 0.76 (from bottom to top) as a function of the P48 crowder concentration for several values of IDP concentration. Panels (**a**,**d**,**g**) in the first column show that the number of IDPs in the Largest Equilibrium Network (LEN) increases with the IDP concentration and that a lower affinity requires a higher crowder concentration before the dense phase appears. The middle column (**b**,**e**,**h**) shows that the separation between the junctions at which IDP binding sites meet is independent of their concentration, and it decreases slowly with increasing crowder concentration. The final column (**c**,**f,i**) shows that the mean number of IDPs binding at the junctions is independent of the IDP concentration but increases weakly with increasing crowder concentration. Note that if the LEN does not exist, the data points lie on the abscissa.

**Figure 4:**
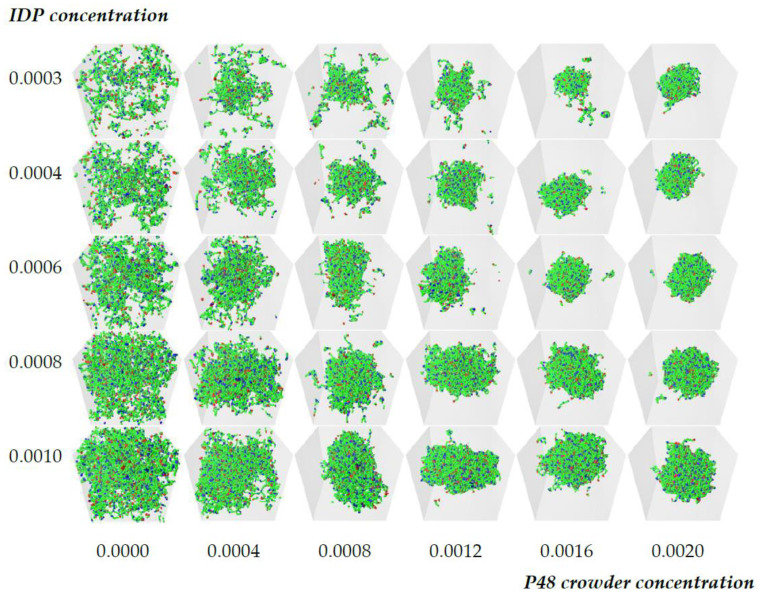
Illustrative grid of snapshots of 6B10 molecules with affinity ε=0.68 in the [Crowder]-[IDP] plane for P48 crowder polymers. As in Figure 2, the rows have constant IDP concentration (from top to bottom): 0.0003, 0.0004, 0.0006, 0.0008, and 0.001, and the crowder concentration increases across the columns (from left to right): 0.0, 0.0004, 0.0008, 0.0012, 0.0016, and 0.002. The binding site attraction is stronger than in Figure 2, but is still too weak to drive phase separation in the absence of the crowder, as seen in the first column.

In Figure 3 we show the quantitative properties of the dense phase of 6B10 IDPs for three values of the affinity, ε=0.6, 0.68, 0.76, that are near the critical value as a function of the crowder concentration. All of the systems in the top row (Figure 3a–c) are phase separated, even in the absence of the crowder, because of their high self-affinity (the grid of snapshots for this affinity, ε=0.76,  are shown in Supplementary Figure S2). Figure 3 panels g–i along the bottom row correspond to the array of simulations in Figure 2, and Figure 3 panels d–f in the middle row correspond to those in Figure 4. The first column shows the number of IDPs that are connected together to form the Largest Equilibrium Network (LEN). This is defined at each sampling point of the simulations as the largest set of IDP molecules that are simultaneously connected by their binding sites. The continual exchange of molecules between the dilute and dense phases causes the LEN to fluctuate over time, and we average many samples to obtain its equilibrium size and properties. We recalculated the LEN for each sample used in the quantitative analysis to minimize the influence of small clusters and surface effects, as described previously [50]. Whereas the size of the LEN for high affinities (Figure 3a) was independent of the crowder concentration, systems with lower affinities (Figure 3d,g) required a higher crowder concentration before the LEN became stable. Note that values of the observables that are near zero means there were too few IDPs in the LEN to calculate the equilibrium averages, i.e., almost all of the IDP molecules are dispersed. The fraction of IDPs in the dense phase increased with the crowder concentration, until it contained all of the IDPs at the highest concentration.

The second column shows the mean junction separation within the condensed phase as a function of the crowder concentration for all of the IDP concentrations studied. The separation was clearly independent of the IDP concentration (and therefore, the droplet size) and the crowder concentration is in the range where the dense phase is stable. Comparing the junction separation in Figure 3b,e,h shows that it was insensitive to the binding site affinity and crowder concentration over the studied range, apart from a slow decrease, which was less than the size of the monomers forming the IDP. Finally, the mean junction mass was insensitive to the IDP concentration, but showed a small systematic increase with increasing crowder concentration (Figure 3c,f,i). Taken together, these results show that IDPs with weaker affinities require higher crowder concentrations to phase separate. The mean junction separation in the dense phase was largely insensitive to the IDP and crowder concentrations, while the number of IDPs binding at the existing junctions increased slowly with increasing crowder concentration.

### 3.3. Dense Phase Structure Is Partially Decoupled from the Crowder Molecular Weight and Enthalpic Repulsion from the IDPs

#### 3.3.1. The Phase Boundary but Not the Dense Phase Structure Varies with the Crowder Volume Fraction

The previous section showed that the structural organization of the IDPs in the equilibrium dense phase was independent of the IDP concentration (once the dense phase is stable), and was only weakly dependent on the crowder concentration. Next, we probed the dependence of this structure on the molecular weight of the crowder. Because we expected the reduced volume fraction of shorter crowders to impose a smaller osmotic pressure on the IDPs, we increased the IDP affinity from ε=0.6 to ε=0.68. The baseline results using the P48 crowders are shown in Figure 4. The first column shows that the higher self-affinity was still too weak to drive phase separation in the absence of a crowder, but the IDPs phase separated with increasing crowder concentration, as observed in Figure 2. 

**Figure 5:**
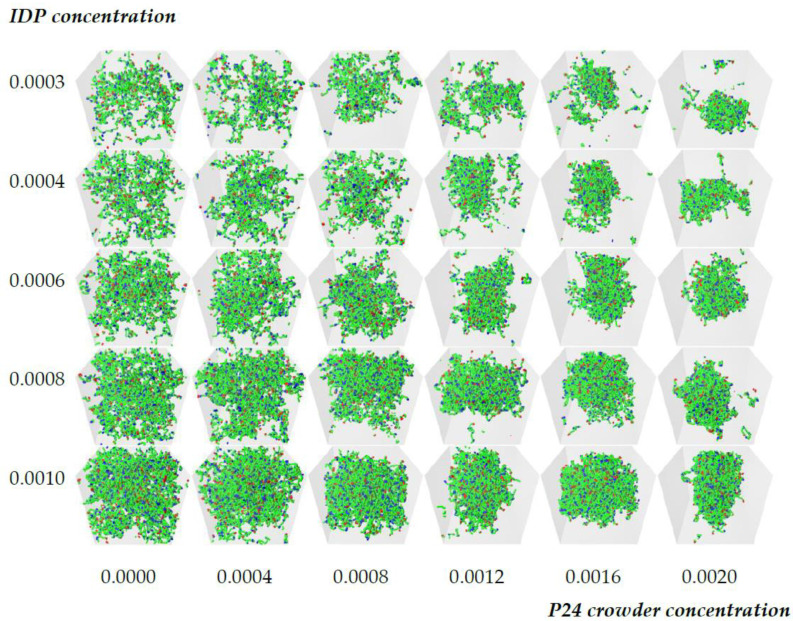
Qualitative effect of reducing the length of the crowder molecules on the dense phase stability of 6B10 molecules with constant affinity ε=0.68. Replacing P48 crowders with P24 ones with the same number fraction halves the monomer volume fraction of the crowders. The IDPs phase separate at higher crowder concentrations, and more IDPs are in the dilute phase compared to Figure 4.

We then repeated these simulations with crowding polymers whose size was reduced from 48 to 24 monomers, and we kept their number fractions for each grid element the same as before (Figure 5). This effectively reduced their volume fraction by a factor of two. (Supplementary Figure S3 shows the grid with the visible P24 crowder molecules.) Comparing Figure 4 and Figure 5 shows that the phase boundary shifted to a higher crowder concentration in the presence of the P24 crowders compared to the P48 ones because more IDPs moved from the dense to the dilute phase, but the droplet still formed. 

Replacing P48 crowders with P24 ones with the same number fraction halves the monomer volume fraction of the crowders. The IDPs phase separate at higher crowder concentrations, and more IDPs are in the dilute phase compared to Figure 4.

**Figure 6:**
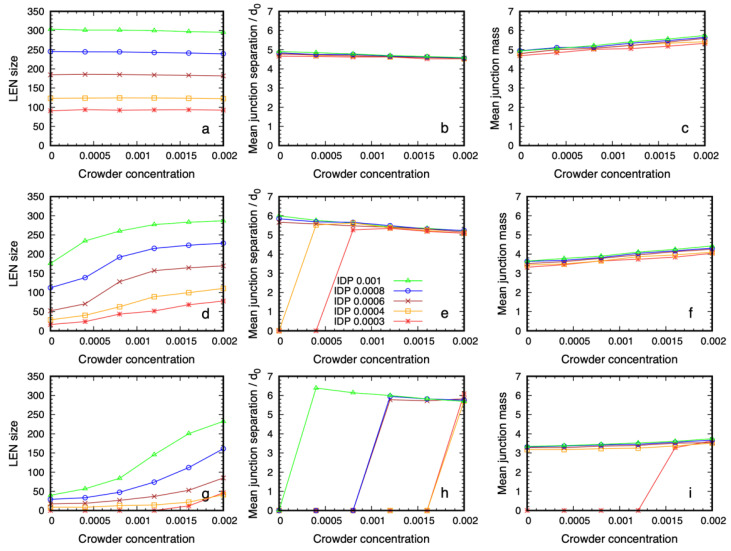
Structural data on the dense phase of 6B10 IDPs with affinities ε=0.6, 0.68, 0.76 (from bottom to top) as a function of the P24 crowder concentration for several values of IDP concentration. Panels (**a**,**d**,**g**) in the first column show that the number of IDPs in the Largest Equilibrium Network (LEN) increases with increasing IDP concentration and that a lower affinity requires a higher crowder concentration before the dense phase appears. The middle column (**b**,**e**,**h**) shows that the separation between the junctions at which IDP binding sites meet is independent of the IDP concentration, and decreases slowly with increasing crowder concentration. The final column (**c**,**f**,**i**) shows that the mean number of IDPs binding at the junctions is also independent of the IDP concentration but increases weakly with increasing crowder concentration. Note that if the LEN does not exist, the data points lie on the abscissa.

We measured the quantitative structure of the dense phase in the presence of P24 crowders, and we show the results in Figure 6. For the IDPs with the highest affinity, the dense phases were almost identical for the two types of crowding polymer. When the affinity was lowered, the most noticeable effect was that a higher crowder concentration was needed to drive the phase separation of the IDPs, which is intuitively expected. Once the dense phase appears, its quantitative structure was only weakly dependent on the crowding polymer length. For the lowest affinity case (Figure 6 panels g–i), the mean junction separation increased by approximately 20%, and the mean junction mass decreased by 10–15%. This shows that the spatial organization of the IDPs inside the dense phase was largely decoupled from the molecular weight of the crowders when their volume fraction was constant.

**Figure 7:**
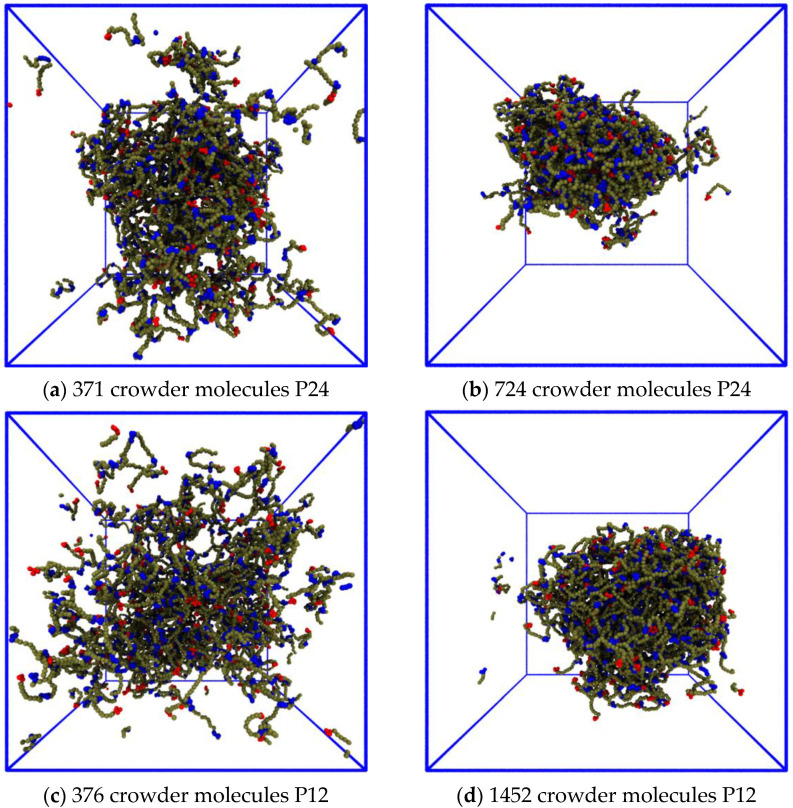
Effects of reducing the length of the crowder molecules on the dense phase stability. (**a**) Replacing P48 crowders with P24 ones at a constant number fraction halves the volume fraction. The droplet remains phase separated, but more IDPs move into the dilute phase. (**b**) The droplet becomes stable again when the number fraction of P24 crowders is increased by a factor of 2, thereby restoring their volume fraction to the original value. (**c**) The droplet dissolves when the P48 crowders are replaced with P12 ones at constant number fraction. (**d**) The droplet is stable again when the number fraction of P12 crowders is increased by a factor of 4, thereby restoring their volume fraction.

Next, we replaced the P24 crowders with P12 ones and qualitatively checked whether the dense phase remained stable. We selected the system in the third row and fourth column of Figure 4 as a typical case. The IDP and crowder number fractions were 0.0006 and 0.0012, respectively, (corresponding to 180 IDPs and 361 crowders, respectively). The conservative repulsive parameter between the crowder monomers and IDP monomers was kept at $a_{Px}=80$, where × stands for all of the bead types, except for the solvent (cp. Table 1). We performed separate simulations using the P24 crowders, with number fractions of 0.0012 and 0.0024, and P12 crowders, with number fractions 0.0012 and 0.0048. Figure 7a,b shows that the P24 crowders at the same number fraction as that of the P48 ones reduced the stability of the droplet, and more IDPs migrated to the dilute phase. Keeping the P24 volume fraction equal to that of the P48 crowder restored the droplet’s stability. Supplementary Movie SM3 shows the evolution of the P24 system over 10^6^ time steps for the same number fraction and same volume fraction. Figure 7c,d shows the same result for P12 crowders; the droplet dissolved due to the reduced crowding effect of the P12 molecules at the same number fraction, but its stability was restored when their number fraction was increased by a factor of four. Supplementary Movie SM4 shows the evolution of the P12 system over 10^6^ time steps for the same number fraction and volume fraction.

#### 3.3.2. Reducing the Enthalpic Repulsion of the IDPs from the Crowder Molecules Leaves the Dense Phase Stable

**Figure 8:**
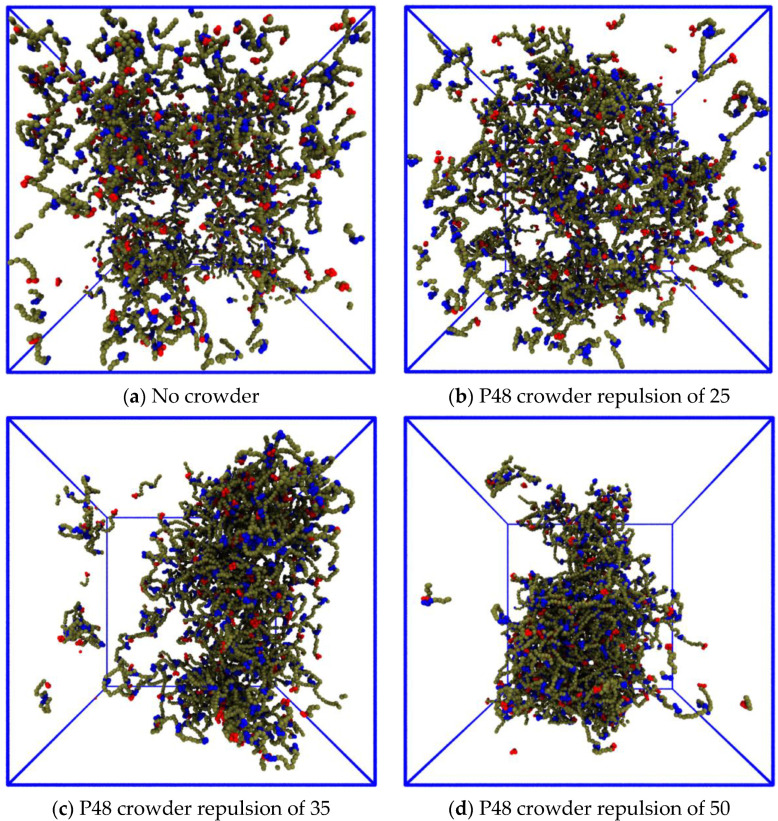
Effect of reducing the repulsive conservative force between the P48 crowder polymers and the IDPs. Each panel has the same number fraction of 6B10 IDPs with binding site affinity ε=0.68. (**a**) The IDPs do not phase separate in the absence of crowder polymers. (**b**) The same repulsion between the crowders and the IDPs as their self-repulsion does not lead to phase separation. (**c**) A slightly greater repulsion enhances phase separation. (**d**) Stronger repulsion leads to complete phase separation.

Next, we investigated the impact of reducing the enthalpic repulsion between the IDPs and crowder molecules on the phase separation. We again used the system in the third row and fourth column of Figure 4. The baseline repulsion of $a_{Px}=80$ between the crowder monomers and all of the other (non-solvent) bead types x was reduced to $a_{Px}=50,\;35,\;25$ at 500,000 steps in independent simulations. Figure 8 shows snapshots of the systems after 2 × 10^6^ time steps, which ensured that the IDPs had time to disperse. The IDPs do not phase separate for this combination of concentration and affinity in the absence of the crowder (Figure 8a) nor when the repulsion between the crowder and IDP polymers was the same as the IDP self-repulsion (Figure 8b). The crowding agent was able to assist the phase separation when its repulsion from the IDPs was greater than $a_{Px}=35$, which is a value that is not much higher than that of the IDP’s self-repulsion (Figure 8c,d). This shows that the phase separation was not greatly sensitive to the enthalpic repulsion between the IDPs and the crowding agents as long as it was above a minimal threshold. (Supplementary Movie SM5 shows the evolution of the systems shown in Figure 8 panels b and d for the second 10^6^ time steps).

## 4. Discussion

The cellular cytoplasm is a complex, crowded fluid, and understanding the effects of this environment on the phase separation of disordered proteins into biomolecular condensates is a challenging task. Most in vitro assays and computational models focus on a single protein, and their relevance to living cells is an open question [39]. A full statistical mechanical theory of the phase behavior of a self-associating polymer appears extremely difficult to construct, although attempts of various kinds have been made [65,66,67]. In the presence of a polymeric crowding agent, it becomes (probably) impossible. Yet experiments show that a crowded environment can influence the appearance of pathological fibrillar states within biomolecular condensates [68,69,70,71]. It has also been found that the material properties of BCs are important for their cellular functions [34,37] and disordered protein domains can exert steric pressure on the cellular membranes [72]. Predicting the response of condensed phases of IDPs to the crowdedness of their environment is an important step in understanding their functions in health and possible role in treating diseases [29,30,73].

Experiments on full-length FUS and its N-terminal low-complexity domain (FUS-LC) have shown that their dense phase is highly solvated [42,43]. The flexible IDPs form transient attractive contacts along their length and retain large conformational fluctuations in the dense phase. Their phase separation (at least in the case of FUS-LC, which is almost uncharged and has fewer than 25% hydrophobic residues) is therefore unlike oil–water phase separation, and we do not expect mean field theories (including Flory-Huggins theory) to describe their phase separation correctly [74]. Recent computational modelling suggested that conformational fluctuations are an important driver of the transition [49,50,53,75,76].

In the present work, we implemented a novel workflow to leverage multiple simultaneous simulations to explore the two-dimensional phase space of an IDP in the presence of a crowding agent. Interestingly, an analogous experimental microfluidic platform was recently used to rapidly scan multidimensional phase diagrams [77]. Massively-parallel tools such as these are needed to explore the increasingly complex experimental systems, such as ternary systems with and without an inert crowding agent [78].

We used our workflow to explore the effects of adding a polymeric crowding agent on the phase separation of a model IDP using coarse-grained simulations. This provides a tractable model system to explore the range of possible responses of a BC to a crowded environment. The phase behavior of the model proteins is controlled by their molecular weight, and the number, distribution, and affinity of their sticky sites. In the absence of a crowding agent, they phase separate into coexisting dilute and dense phases when the affinity of their sticky sites is sufficiently strong, form a space-filling network without phase separation for lower affinities, and remain dispersed when their attractive interactions are weak or absent [45,50,56]. The structural properties of the dense phase in coarse-grained models have been shown to be modulated by specific and non-specific molecular interactions [79,80,81,82]. 

When a polymeric crowding agent was added to IDPs in solution, we observed the following effects: (1) if the IDP affinity was just below the value at which spontaneous phase separation occurred, the crowder shifted the transition so that the IDPs phase separated into a dense phase; (2) the observed dense phase had a similar spatial structure to that of spontaneously formed droplets of the same IDPs with higher affinity. The formation of the model biomolecular condensate is therefore coupled to the crowdedness of its environment. This is in good agreement with recent experimental results of André et al. that showed that crowding lowered the dilute phase boundary and increased the dense phase concentration in the phase diagram of NPM1-RNA condensates [83]. Alshareedah et al. recently found that the mobility of the peptide in a heterogeneous dense phase of peptide + ssDNA scaled with the flow activation energy of the condensate [84]. This finding was predicted by our simulations in previous work, which that showed that the fluidity of the model IDPs in their dense phase decreases with increasing binding affinity, and also decreases when the same number of sticky sites are spaced farther apart on the model IDPs [50]. This suggests that the mobility of the macromolecules in a biomolecular condensate is predominantly controlled by their binding/unbinding, which was controlled in our simulations by the affinity parameter. Our results also predict that the internal structure of the model condensate is only weakly affected by the crowded environment over the studied concentration range. This may explain how WNK1 kinase responds to osmotic stress in cells by condensing into a functional biomolecular condensate [15]. It is important to point out here that this structural resistance does not imply that the mobility of the molecules in the dense phase is independent of the crowding, only that the structural arrangement of the molecules is resistant to deformation. We note here that our simulations probed the situation in which crowding polymers exert only steric and entropic forces on the IDPs in a concentration range that is similar to that of the cellular cytoplasm. The densest system we simulated had a crowder concentration of 120 mM, which is comparable to the estimates of 100–450 mg/mL for cellular cytoplasm [1,2]. This is a simpler case than that of the common crowding agent PEG, which strongly modifies the water activity in its environment [3]. Indeed, PEG has recently been observed not only to assist phase separation of the protein NPM1 with rRNA, but also to be concentrated inside the condensate [83]. This case could be explored by extending our approach to include attractive interactions between the IDPs and the PEG polymers. Our results suggest that a cell may partition the cytoplasm into regions in which the phase boundary of a biomolecular condensate is tuned by the local concentration of external macromolecules (i.e., non-partitioning crowding agents), while its interior is (partially) decoupled from the cytoplasmic composition, thereby producing a stable internal fluid environment.

The spatial arrangement of the IDPs in the crowding-assisted dense phase was similar to that observed previously for the same IDPs with higher affinity that spontaneously phase separate. The structure of the model BC was therefore not strongly dependent on the strength of the attractive interactions between the constituent IDPs. We also observed that the model condensate still formed when the repulsion between the IDPs and the crowder was substantially lowered and when we reduced the crowder molecular weight, keeping their volume fraction fixed. 

We hypothesize that the internal degrees of freedom of the IDPs, which are defined by their molecular sequence, are responsible for the robust structure of their dense phase. The punctate nature of their binding sites creates a spatial network of weak junctions connected by fluctuating spring-like lengths of the backbone that resist deformation when the osmotic pressure due to the surrounding fluid increases. Our results are in good agreement with the recent results of Alshareedah et al. who found that the viscoelastic properties of in vitro BCs were controlled by the combination of enthalpic (sequence-encoded interchain interactions) and entropic (fluctuations of the polymer chain-like IDPs) factors [84]. This may be compared to a single-component lipid bilayer vesicle, in which the membrane is stabilized by the strong hydrophobic repulsion of the lipid tails from the aqueous solvent. Adding cholesterol modifies the internal degrees of freedom of the lipids (chain ordering) and gives rise to a new phase: the liquid ordered phase. We speculate that as for the liquid ordered phase, biomolecular condensates rely on the modification of their internal degrees of freedom to create robust microenvironments, even in the presence of changing external concentrations of crowding macromolecules. 

Computational modelling provides a relatively inexpensive tool for exploring simplified representations of an experimental system, but it suffers from its own complexity issues, namely, that the number of parameters increases rapidly with increasing numbers of molecular species. Our exploration of the response of a model biomolecular condensate to the presence of a crowding agent illustrates the following powerful workflow for studying models with high-dimensional parameter spaces: (1) generate many examples of a system at different points in its parameter space simultaneously, and (2) rapidly compare the examples by viewing the data in a large grid of snapshots taken from different parameter space points, and identify the interesting regions. Further progress will likely involve using AI-enabled pattern recognition of the graphical arrays to identify the most interesting regions and direct costly computer resources towards the most efficient pathway for exploration.

## 5. Conclusions

Biomolecular condensates are widely viewed as providing a localized environment for cells to spatiotemporally segregate their biochemistry, despite not being surrounded by a phospholipid membrane. The exchange of proteins between the dense and dilute phases and the crowding effect of other macromolecular species in the cytoplasm would appear to undermine their function. Our coarse-grained molecular simulations predict that a repulsive macromolecular crowding agent is able to drive a dilute solution of IDPs across their phase boundary into a dense phase, even when their attractive self-interactions are too weak to drive phase separation alone. However, crucially, the spatial arrangement of proteins within the resulting BC changes minimally from that of spontaneously phase separating IDPs. These results were also found when the molecular weight of the crowding polymer was reduced (by a factor of two), providing that their volume fraction was maintained, and when the crowder–IDP repulsion was reduced significantly. The dense phase structure was therefore predicted to be insensitive to the precise composition of its crowded environment. We propose that this arises because the pattern of attractive domains connected by entropic spring-like linkers along the IDPs creates a fluid, three-dimensional, network structure that resists deformation by the crowding polymers. Our results suggest that a cell may use the local cytoplasmic concentration of macromolecules to tune the formation of BCs, and that they may in turn sense, and respond to, changes in their environment. 

The effects of crowding on the model condensate are in good agreement with the experiments, and we expect that tailoring this workflow for multicomponent biomolecular condensates will help researchers to generate novel hypotheses that span the complexity gap between simplified computational models and biologically relevant systems.

When the self-attraction of the IDPs is removed, a sufficiently high concentration of the crowder drives a Flory–Huggins-like demixing of the IDPs and crowder polymers, which visually resembles the model BC. However, the dense phase has no connected network structure, because the IDPs are unable to form transiently stable junctions. When the IDP self-attraction is turned back on, the model BC reforms with the same internal structure as before (see Supplementary Movie SM6 and Supplementary Movie SM7). This suggests that a functional BC has a different spatial molecular arrangement to those of the same proteins compressed into a small volume solely by steric crowding.

Our prediction of two visually similar dense phases, only one of which possesses a robust spatially organized structure, implies that the observation of a demixing transition in computational studies of IDP phase separation is insufficient to conclude that their dense phase is a good model of a biomolecular condensate. We believe that the crucial question is whether the spatial structure that we observed, which has not been reported in the literature by other groups, can be found in experiments of biological condensates. Experimental tests of this prediction by positionally mutating key residues in the IDP FUS and exploring the consequences for its phase behavior in the presence of a crowder are under way.

## Figures and Tables

**Table 1:** Bead–bead conservative force parameters $a_{ij}$ (in units of $k_{B}T/d_{0}$ ) for all bead types. The table is symmetrical.

$a_{ij}$	W	E	B	F	P
**W**	25				
**E**	25	a_EE_			
**B**	23	25	25		
**F**	25	a_EE_	25	a_EE_	
**P**	25	80	80	80	80

## Data Availability

Simulation datasets are available on reasonable request to the corresponding author.

## Supplementary Materials

The following supporting information can be downloaded at: https://www.mdpi.com/article/10.3390/biology12020181/s1, Figure S1: Grid in Figure 2 with visible crowder polymers; Figure S2: Grid of IDPs of type 6B10 with high affinity ε=0.76; Figure S3: Grid in Figure 5 with visible crowder polymers; Video SM1: Evolution of the system in grid element 3,4 in Figure 2 with affinity removed at time 500,000. Video SM2: Evolution of the system in grid element 3,6 in Figure 2 with affinity removed at time 500,000. Video SM3: Evolution of the system in grid element 3, 4 in Figure 5 with P24 crowder polymers at the original number fraction (left) and volume fraction (right). Video SM4: Evolution of the system in grid element 3, 4 in Figure 5 with P12 crowder polymers at the original number fraction (left) and volume fraction (right). Video SM5: Evolution of the system in grid element 3, 4 in Figure 2 with IDP/crowder repulsion removed (aPX = 25) (left) and reduced (aPX = 50) (right). Video SM6: Evolution of grid element 3, 6 in Figure 2 with affinity removed at time 500,000. Video SM7: Simulation restarted from the end of SM6 with the IDP affinity restored to ε=0.68 at time zero.

## References

[1] Ellis R.J., Minton A.P. (2003). Join the crowd. Nature.

[2] Feig M., Yu I., Wang P.-H., Nawrocki G., Sugita Y. (2017). Crowding in Cellular Environments at an Atomistic Level from Computer Simulations. J. Phys. Chem. B.

[3] Tyrrell J., Weeks K.M., Pielak G.J. (2015). Challenge of Mimicking the Influences of the Cellular Environment on RNA Structure by PEG-Induced Macromolecular Crowding. Biochemistry.

[4] Wilson E.B. (1899). The Structure of Protoplasm. Science.

[5] Hyman A.A., Brangwynne C.P. (2011). Beyond Stereospecificity: Liquids and Mesoscale Organization of Cytoplasm. Dev. Cell.

[6] Mitrea D.M., Kriwacki R.W. (2016). Phase separation in biology; functional organization of a higher order. Cell Commun. Signal..

[7] Boeynaems S., Alberti S., Fawzi N.L., Mittag T., Polymenidou M., Rousseau F., Schymkowitz J., Shorter J., Wolozin B., van den Bosch L. (2018). Protein Phase Separation: A New Phase in Cell Biology. Trends Cell Biol..

[8] Lyon A.S., Peeples W.B., Rosen M.K. (2020). A framework for understanding the functions of biomolecular condensates across scales. Nat. Rev. Mol. Cell Biol..

[9] Banani S.F., Lee H.O., Hyman A.A., Rosen M.K. (2017). Biomolecular Condensates: Organizers of Cellular Biochemistry. Nat. Rev. Mol. Cell Biol..

[10] Holehouse A.S., Pappu R.V. (2018). Functional Implications of Intracellular Phase Transitions. Biochemistry.

[11] Bratek-Skicki A., Pancsa R., Meszaros B., Van Lindt J., Tompa P. (2020). A guide to regulation of the formation of biomolecular condensates. FEBS J..

[12] Zhang J.Z., Mehta S., Zhang J. (2021). Liquid–liquid phase separation: A principal organizer of the cell’s biochemical activity architecture. Trends Pharmacol. Sci..

[13] Lee R., Buljan M., Lang B., Weatheritt R.J., Daughdrill G.W., Dunker A.K. (2014). Classification of Intrinsically Disordered Regions and Proteins. Chem. Rev..

[14] Oldfield C.J., Dunker A.K. (2014). Intrinsically Disordered Proteins and Intrinsically Disordered Protein Regions. Annu. Rev. Biochem..

[15] Boyd-Shiwarski C.R., Shiwarski D.J., Griffiths S.E., Beacham R.T., Norrell L., Morrison D.E., Wang J., Mann J., Tennant W., Anderson E.N. (2022). WNK kinases sense molecular crowding and rescue cell volume via phase separation. Cell.

[16] Pastore A., Temussi P.A. (2022). Crowding revisited: Open questions and future perspectives. Trends Biochem. Sci..

[17] Nedelsky N.B., Taylor J.P. (2019). Bridging biophysics and neurology: Aberrant phase transitions in neurodegenerative disease. Nat. Rev. Neurol..

[18] Alberti S., Hyman A.A. (2021). Biomolecular condensates at the nexus of cellular stress, protein aggregation disease and ageing. Nat. Rev. Mol. Cell Biol..

[19] Alberti S., Hyman A.A. (2016). Are Aberrant Phase Transitions a Driver of Cellular Aging?. Bioessays.

[20] Ranganathan S., Shakhnovich E. (2022). The physics of liquid-to-solid transitions in multi-domain protein condensates. Biophys. J..

[21] Agarwal A., Mukhopadhyay S. (2021). Prion Protein Biology Through the Lens of Liquid-Liquid Phase Separation. J. Mol. Biol..

[22] Bracha D., Walls M.T., Brangwynne C.P. (2019). Probing and engineering liquid-phase organelles. Nat. Biotechnol..

[23] Dzuricky M., Rogers B.A., Shahid A., Cremer P.S., Chilkoti A. (2020). De novo engineering of intracellular condensates using artificial disordered proteins. Nat. Chem..

[24] Heidenreich M., Georgeson J.M., Locatelli E., Rovigatti L., Nandi S.K., Steinberg A., Nadav Y., Shimoni E., Safran S.A., Doye J.P.K. (2020). Designer protein assemblies with tunable phase diagrams in living cells. Nat. Chem. Biol..

[25] Garabedian M.V., Wang W., Dabdoub J.B., Tong M., Caldwell R.M., Benman W., Schuster B.S., Deiters A., Good M.C. (2021). Designer membraneless organelles sequester native factors for control of cell behavior. Nat. Chem. Biol..

[26] Hastings R.L., Boeynaems S. (2021). Designer Condensates: A Toolkit for the Biomolecular Architect. J. Mol. Biol..

[27] Mu W., Ji Z., Zhou M., Wu J., Lin Y., Qiao Y. (2021). Membrane-confined liquid-liquid phase separation toward artificial organelles. Sci. Adv..

[28] Qian Z.-G., Huang S.-C., Xia X.-X. (2022). Synthetic protein condensates for cellular and metabolic engineering. Nat. Chem. Biol..

[29] Biesaga M., Frigolé-Vivas M., Salvatella X. (2021). Intrinsically disordered proteins and biomolecular condensates as drug targets. Curr. Opin. Chem. Biol..

[30] Mitrea D.M., Mittasch M., Gomes B.F., Klein I.A., Murcko M.A. (2022). Modulating biomolecular condensates: A novel approach to drug discovery. Nat. Rev. Drug Discov..

[31] Patel A., Mitrea D., Namasivayam V., Murcko M.A., Wagner M., Klein I.A. (2022). Principles and functions of condensate modifying drugs. Front. Mol. Biosci..

[32] Nott Timothy J., Petsalaki E., Farber P., Jervis D., Fussner E., Plochowietz A., Craggs T.D., Bazett-Jones D.P., Pawson T., Forma-Kay J.D. (2015). Phase Transition of a Disordered Nuage Protein Generates Environmentally Responsive Membrane less Organelles. Mol. Cell.

[33] Banani S.F., Rice A.M., Peeples W.B., Lin Y., Jain S., Parker R., Rosen M.K. (2016). Compositional Control of Phase-Separated Cellular Bodies. Cell.

[34] Shin Y., Chang Y.-C., Lee D.S., Berry J., Sanders D.W., Ronceray P., Wingreen N.S., Haataja M., Brangwynne C.P. (2018). Liquid Nuclear Condensates Mechanically Sense and Restructure the Genome. Cell.

[35] Zhao Y.G., Zhang H. (2020). Phase Separation in Membrane Biology: The Interplay between Membrane-Bound Organelles and Membraneless Condensates. Dev. Cell.

[36] Gouveia B., Kim Y., Shaevitz J.W., Petry S., Stone H.A., Brangwynne C.P. (2022). Capillary forces generated by biomolecular condensates. Nature.

[37] Lasker K., Boeynaems S., Lam V., Scholl D., Stainton E., Briner A., Jacquemyn M., Daelemans D., Deniz A., Villa E. (2022). The material properties of a bacterial-derived biomolecular condensate tune biological function in natural and synthetic systems. Nat. Commun..

[38] Wang H.-Y., Chan S.H., Dey S., Castello-Serrano I., Ditlev J.A., Rosen M.K. (2022). Coupling of protein condensates to ordered lipid domains determines functional membrane organization. Biorxiv.

[39] Musacchio A. (2022). On the role of phase separation in the biogenesis of membrane less compartments. EMBO J..

[40] Mittag T., Pappu R.V. (2022). A conceptual framework for understanding phase separation and addressing open questions and challenges. Mol. Cell.

[41] Patel A., Lee H.O., Jawerth L., Maharana S., Jahnel M., Hein M.Y., Stoynov S., Mahamid J., Saha S., Franzmann T.M. (2015). A Liquid-to-Solid Phase Transition of the ALS Protein FUS Accelerated by Disease Mutation. Cell.

[42] Burke K.A., Janke A.M., Rhine C.L., Fawzi N.L. (2015). Residue-by-Residue View of In Vitro FUS Granules that Bind the C-Terminal Domain of RNA Polymerase II. Mol. Cell.

[43] Murthy A.C., Dignon G.L., Kan Y., Zerze G.H., Parekh S.H., Mittal J., Fawzi N.L. (2019). Molecular interactions underlying liquid−liquid phase separation of the FUS low-complexity domain. Nat. Struct. Mol. Biol..

[44] Murthy A.C., Tang W.S., Jovic N., Janke A.M., Seo D.H., Perdikari T.M., Mittal J., Fawzi N.L. (2021). Molecular interactions contributing to FUS SYGQ LC-RGG phase separation and co-partitioning with RNA polymerase II heptads. Nat. Struct. Mol. Biol..

[45] Harmon T.S., Holehouse A.S., Rosen M.K., Pappu R.V. (2017). Intrinsically disordered linkers determine the interplay between phase separation and gelation in multivalent proteins. eLife.

[46] Dignon G.L., Zheng W., Kim Y.C., Best R.B., Mittal J. (2018). Sequence determinants of protein phase behavior from a coarse-grained model. PLOS Comput. Biol..

[47] Dignon G.L., Zheng W., Mittal J. (2019). Simulation methods for liquid–liquid phase separation of disordered proteins. Curr. Opin. Chem. Eng..

[48] Ruff K.M., Pappu R.V., Holehouse A.S. (2019). Conformational Preferences and Phase Behaviour of Intrinsically Disordered Low Complexity Sequences: Insights from Multiscale Simulations. Curr. Op. Struct. Biol..

[49] Benayad Z., von Bülow S., Stelzl L.S., Hummer G. (2020). Simulation of FUS Protein Condensates with an Adapted Coarse-Grained Model. J. Chem. Theory Comput..

[50] Shillcock J.C., Lagisquet C., Alexandre J., Vuillon L., Ipsen J.H. (2022). Model biomolecular condensates have heterogeneous structure quantitatively dependent on the interaction profile of their constituent macromolecules. Soft Matter.

[51] Shillcock J.C., Thomas D.B., Beaumont J.R., Bragg G.M., Vousden M.L., Brown A.D. (2022). Coupling Bulk Phase Separation of Disordered Proteins to Membrane Domain Formation in Molecular Simulations on a Bespoke Compute Fabric. Membranes.

[52] Pyo A.G., Zhang Y., Wingreen N.S. (2022). Surface tension and super-stoichiometric surface enrichment in two-component biomolecular condensates. Iscience.

[53] Weiner B.G., Pyo A.G.T., Meir Y., Wingreen N.S. (2021). Motif-pattern dependence of biomolecular phase separation driven by specific interactions. PLOS Comput. Biol..

[54] Hoogerbrugge P.J., Koelman J.M.V.A. (1992). Simulating Microscopic Hydrodynamic Phenomena with Dissipative Particle Dynamics. EPL Europhys. Lett..

[55] Groot R.D., Warren P.B. (1997). Dissipative particle dynamics: Bridging the gap between atomistic and mesoscopic simulation. J. Chem. Phys..

[56] Shillcock J.C., Brochut M., Chénais E., Ipsen J.H. (2020). Phase behaviour and structure of a model biomolecular condensate. Soft Matter.

[57] Beaumont J.R., Brown A.D., Thomas D.B., Shillcock J.C., Naylor M.F., Bragg G.M., Vousden M., Moore S.W., Flemming S. (2023). An event-driven approach to Dissipative Particle Dynamics. ACM Trans. Parallel Comput..

[58] Brown A.D., Thomas D.B., Reeve J., Tarawneh G., De Gennaro A., Mokhov A., Naylor M., Kazmierski T. (2017). Distributed event-based computing. Proceedings of the ParCo 2017: Parallel Computing is Everywhere.

[59] Español P., Warren P. (1995). Statistical Mechanics of Dissipative Particle Dynamics. EPL Europhys. Lett..

[60] Espagnol P., Warren P.B. (2017). Perspective: Dissipative Particle Dynamics. J Chem Phys..

[61] Shillcock J.C., Lipowsky R. (2002). Equilibrium structure and lateral stress distribution of amphiphilic bilayers from dissipative particle dynamics simulations. J. Chem. Phys..

[62] Shillcock J.C. OSPREY-DPD. Open Source Polymer Research Engine-Dissipative Particle Dynamics. 2020. https://github.com/Osprey-DPD/osprey-dpd.

[63] Wang J., Choi J.-M., Holehouse A.S., Lee H.O., Zhang X., Jahnel M., Maharana S., Lemaitre R., Pozniakovsky A., Drechsel D. (2018). A Molecular Grammar Governing the Driving Forces for Phase Separation of Prion-like RNA Binding Proteins. Cell.

[64] Rubinstein M., Colby R.H. (2003). Polymer Physics.

[65] Zilman A., Tlusty T., Safran S.A. (2002). Entropic networks in colloidal, polymeric and amphiphilic systems. J. Phys. Condens. Matter..

[66] Dudowicz J., Freed K.F. (2012). Lattice cluster theory of associating polymers. I. Solutions of linear telechelic polymer chains. J. Chem. Phys..

[67] Parada G.A., Zhao X. (2018). Ideal reversible polymer networks. Soft Matter.

[68] Wen J., Hong L., Krainer G., Yao Q.-Q., Knowles T.P.J., Wu S., Perrett S. (2021). Conformational Expansion of Tau in Condensates Promotes Irreversible Aggregation. J. Am. Chem. Soc..

[69] Horvath I., Kumar R., Wittung-Stafshede P. (2021). Macromolecular crowding modulates α-synuclein amyloid fiber growth. Biophys. J..

[70] Linsenmeier M., Faltova L., Palmiero U.C., Seiffert C., Küffner A.M., Pinotsi D. (2022). The interface of condensates of the hnRNPA1 low complexity domain promotes formation of amyloid fibrils. BioRxiv.

[71] Küffner A.M., Linsenmeier M., Grigolato F., Prodan M., Zuccarini R., Palmiero U.C., Faltova L., Arosio P. (2021). Sequestration within biomolecular condensates inhibits Aβ-42 amyloid formation. Chem. Sci..

[72] Houser J.R., Cho H.W., Hayden C.C., Yang N.X., Wang L., Lafer E.M., Thirumalai D., Stachowiak J.C. (2022). Molecular mechanisms of steric pressure generation and membrane remodeling by disordered proteins. Biophys. J..

[73] Boija A., Klein I.A., Young R.A. (2021). Biomolecular Condensates and Cancer. Cancer Cell.

[74] Kar M., Dar F., Welsh T.J., Vogel L.T., Kühnemuth R., Majumdar A., Krainer G., Franzmann T.M., Alberti S., Seidel C.A.M. (2022). Phase-separating RNA-binding proteins form heterogeneous distributions of clusters in subsaturated solutions. Proc. Natl. Acad. Sci. USA.

[75] Tesei G., Schulze T.K., Crehuet R., Lindorff-Larsen K. (2021). Accurate model of liquid–liquid phase behavior of intrinsically disordered proteins from optimization of single-chain properties. Proc. Natl. Acad. Sci. USA.

[76] Farag M., Cohen S.R., Borcherds W.M., Bremer A., Mittag T., Pappu R.V. (2022). Condensates formed by prion-like low-complexity domains have small-world network structures and interfaces defined by expanded conformations. Nat. Commun..

[77] Arter W.E., Qi R., Erkamp N.A., Krainer G., Didi K., Welsh T.J., Acker J., Nixon-Abell J., Qamar S., Guillén-Boixet J. (2022). Biomolecular condensate phase diagrams with a combinatorial microdroplet platform. Nat. Commun..

[78] Bai Q., Liu Z., Chen J., Liang D. (2023). Crowded Environment Regulates the Coacervation of Biopolymers via Nonspecific Interactions. Biomacromolecules.

[79] Harmon T.S., Holehouse A.S., Pappu R.V. (2018). Differential Solvation of Intrinsically Disordered Linkers Drives the Formation of Spatially Organised Droplets in Ternary Systems of Linear Multivalent Proteins. New J. Physics..

[80] Alshareedah I., Moosa M.M., Pham M., Potoyan D.A., Banerjee P.R. (2021). Programmable viscoelasticity in protein-RNA condensates with disordered sticker-spacer polypeptides. Nat. Commun..

[81] Holehouse A.S., Ginell G.M., Griffith D., Böke E. (2021). Clustering of Aromatic Residues in Prion-like Domains Can Tune the Formation, State, and Organization of Biomolecular Condensates. Biochemistry.

[82] Das S., Muthukumar M. (2022). Microstructural Organization in α-Synuclein Solutions. Macromolecules.

[83] André A.A., Yewdall N.A., Spruijt E. (2022). Crowding-induced phase separation and gelling by co-condensation of PEG in NPM1-rRNA condensates. bioRxiv.

[84] Alshareedah I., Singh A., Quinn A., Banerjee P.R. (2022). Determinants of Viscoelasticity and Flow Activation Energy in Biomolecular Condensates. bioRxiv.

[85] Humphrey W., Dalke A., Schulten K. (1996). VMD: Visual molecular dynamics. J. Mol. Graph..

[86] Schneider C.A., Rasband W.S., Eliceiri K.W. (2012). NIH Image to ImageJ: 25 Years of image analysis. Nat. Methods.

