# Supplementary material for: Macromolecular Crowding Is Surprisingly Unable to Deform the Structure of a Model Biomolecular Condensate
Source: Biology (Basel). 2023 Jan 25;12(2):181. doi: 10.3390/biology12020181 (PMC9952705; doi:10.3390/biology12020181)
Supplement: Supplementary file 1 [file biology-12-00181-s001.zip › Shillcock-ESI.pdf]

Macromolecular crowding is surprisingly unable to deform the structure of a model biomolecular condensate

Julian C. Shillcock [1,*], David B. Thomas [2], John H. Ipsen [3] and Andrew D. Brown [2]

[1] Blue Brain Project and Laboratory of Molecular and Chemical Biology of Neurodegeneration, Ecole polytechnique fédérale de Lausanne, CH-1015 Lausanne, Switzerland; julian.shillcock@epfl.ch
[2] Department of Electronics and Computer Science, University of Southampton, Highfield, Southampton, SO17 1BJ, U.K
[3] Department of Physics, Chemistry and Pharmacy, University of Southern Denmark, Campusvej 55, DK-5230 Odense M, Denmark

* Correspondence: julian.shillcock@epfl.ch; Tel.: +41 21 693 9679

Supplementary Material

Figure S1 Grid of snapshots for 6B10 IDPs with affinity $\varepsilon = 0.6$ in the presence of P48 crowder molecules in a simulation box of size $(48d_0)^3$. It is equivalent to Figure 2 in the main text but the crowder polymers are visible.

Figure S2 Grid of snapshots for 6B10 IDPs with affinity $\varepsilon = 0.76$ in the presence of P48 crowder molecules in a simulation box of size $(48d_0)^3$. It shows that IDPs with this affinity are able to phase separate even in the absence of the crowding polymers.

Figure S3 Grid of snapshots for 6B10 IDPs with affinity $\varepsilon = 0.68$ in the presence of P48 crowder molecules in a simulation box of size $(48d_0)^3$. It is equivalent to Figure 5 in the main text but the crowder polymers are visible.

Supplementary Movie SM1 Evolution of a system containing a number fraction 0.0006 and 0.0012 for the IDP and crowder polymers respectively. This corresponds to grid element 3,4 in Figure 2. The IDP affinity is initially $\varepsilon = 0.68$ and the polymer types are randomly distributed throughout the simulation box. The system demixes to form a dense IDP phase surrounded by the crowding agent. At the mid-point of the

simulation (500,000 steps), the IDP self-affinity is removed $\varepsilon = 0$, and the dense phase dissolves. (The crowder polymers are made invisible soon after the start for clarity.)

Supplementary Movie SM2 Evolution of a system containing a number fraction 0.0006 and 0.002 for the IDP and crowder polymers respectively. This corresponds to grid element 3,6 in Figure 2. The IDP affinity is initially $\varepsilon = 0.68$ and the polymer types are randomly distributed throughout the simulation box. The system demixes to form a dense IDP phase surrounded by the crowding agent. At the mid-point of the simulation (500,000 steps), the IDP self-affinity is removed $\varepsilon = 0$, but the IDPs remain compressed by repulsion from the crowder polymers. (The crowder polymers are made invisible soon after the start for clarity.)

Supplementary Movie SM3 Evolution of two systems containing a number fraction 0.0006 for the IDP and 0.0012 (left) and 0.0024 (right) for the P24 crowder polymers respectively. The left-hand movie corresponds to grid element 3,4 in Figure 5. The IDP affinity is $\varepsilon = 0.68$. Reducing the molecular weight of the crowders from P48 to P24 shifts the system so that more IDPs are in the dilute phase. Increasing the number fraction by a factor of 2, so as to maintain the original crowder volume fraction, restores the dense phase.

Supplementary Movie SM4 Evolution of two systems containing a number fraction 0.0006 for the IDP and 0.0012 (left) and 0.0048 (right) for the P12 crowder polymers respectively. The IDP affinity is $\varepsilon = 0.68$. Reducing the molecular weight of the crowders from P48 to P12 shifts the system even more towards the mixed phase. Increasing the number fraction by a factor of 4, so as to maintain the original crowder volume fraction, restores the dense phase.

Supplementary Movie SM5 Evolution of the two systems shown in Figure 8 b (left), and 8 d (right) after one million time steps to allow them to reach equii.brium after

reducing the crowder polymer repulsion. The systems both contain number fractions 0.0006 and 0.0012 of the IDP and crowder polymers. The IDP self-affinity is $\varepsilon = 0.68$, but the repulsion between the IDPs and crowder polymers is removed in the left movie (a_{PX} = 25) and reduced in the right movie (a_{PX} = 50). The dense phase disappears in the first case, but remains even for the reduced repulsion.

Supplementary Movie SM6 Evolution of the system in grid element 3,6 of Figure 2 for one million time steps. When the IDP self-affinity is removed $\varepsilon = 0$ at the mid-point of the simulation (500,000 steps), the dense phase remains loosens but remains phase separated. The simulation is continued in Supplementary movie SM7.

Supplementary Movie SM7 Simulation of one million steps restarted from the end of Supplementary movie SM6. The IDP self-affinity is restored at time zero to its original value $\varepsilon = 0.68$. The dense phase reforms and is again the equilibrium state.
