# Supplementary figures and images for: Macromolecular Crowding Is Surprisingly Unable to Deform the Structure of a Model Biomolecular Condensate
Source: Biology (Basel). 2023 Jan 25;12(2):181. doi: 10.3390/biology12020181 (PMC9952705; doi:10.3390/biology12020181)

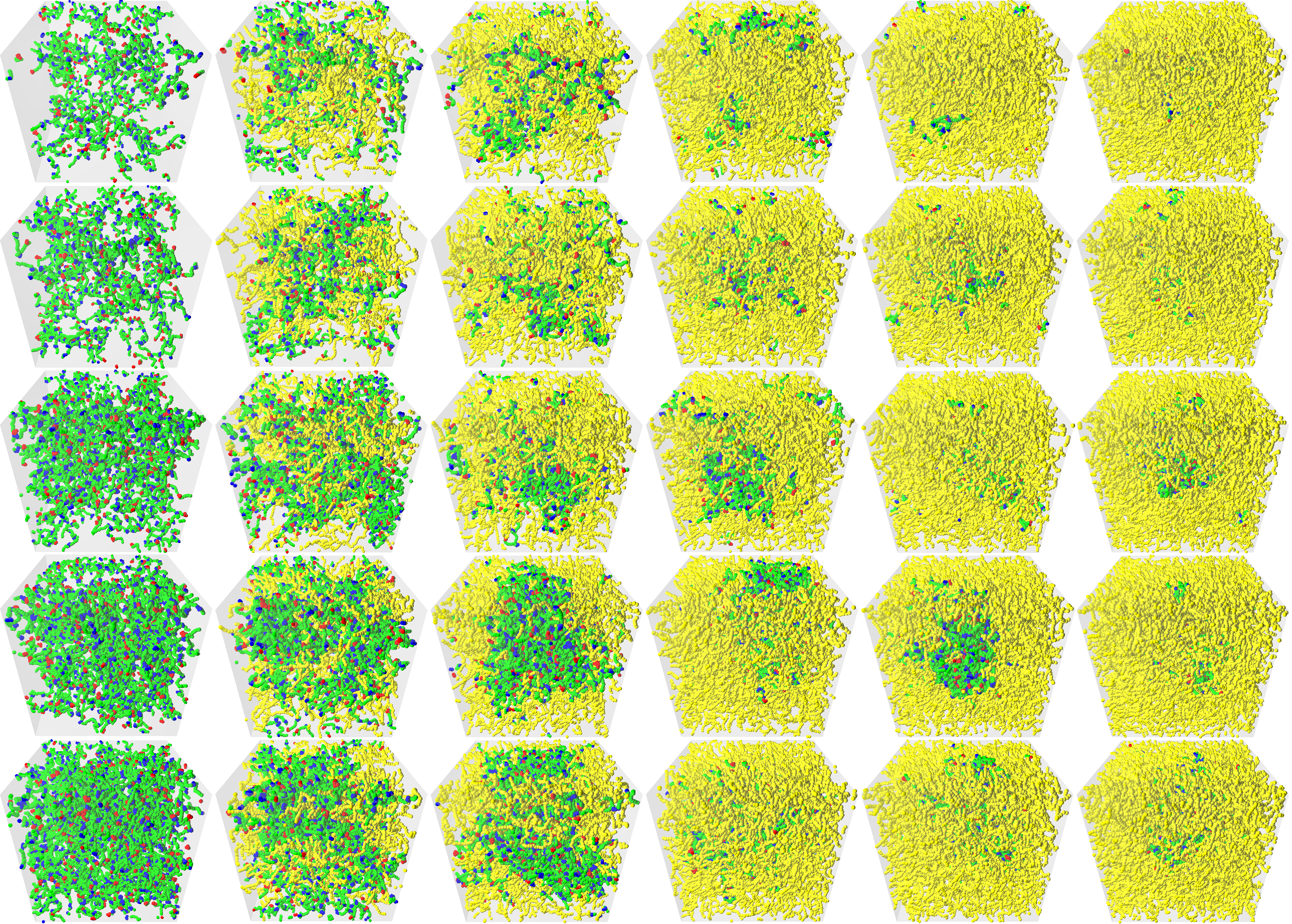

Supplement: Supplementary file 1 [file biology-12-00181-s001.zip › S1.png]

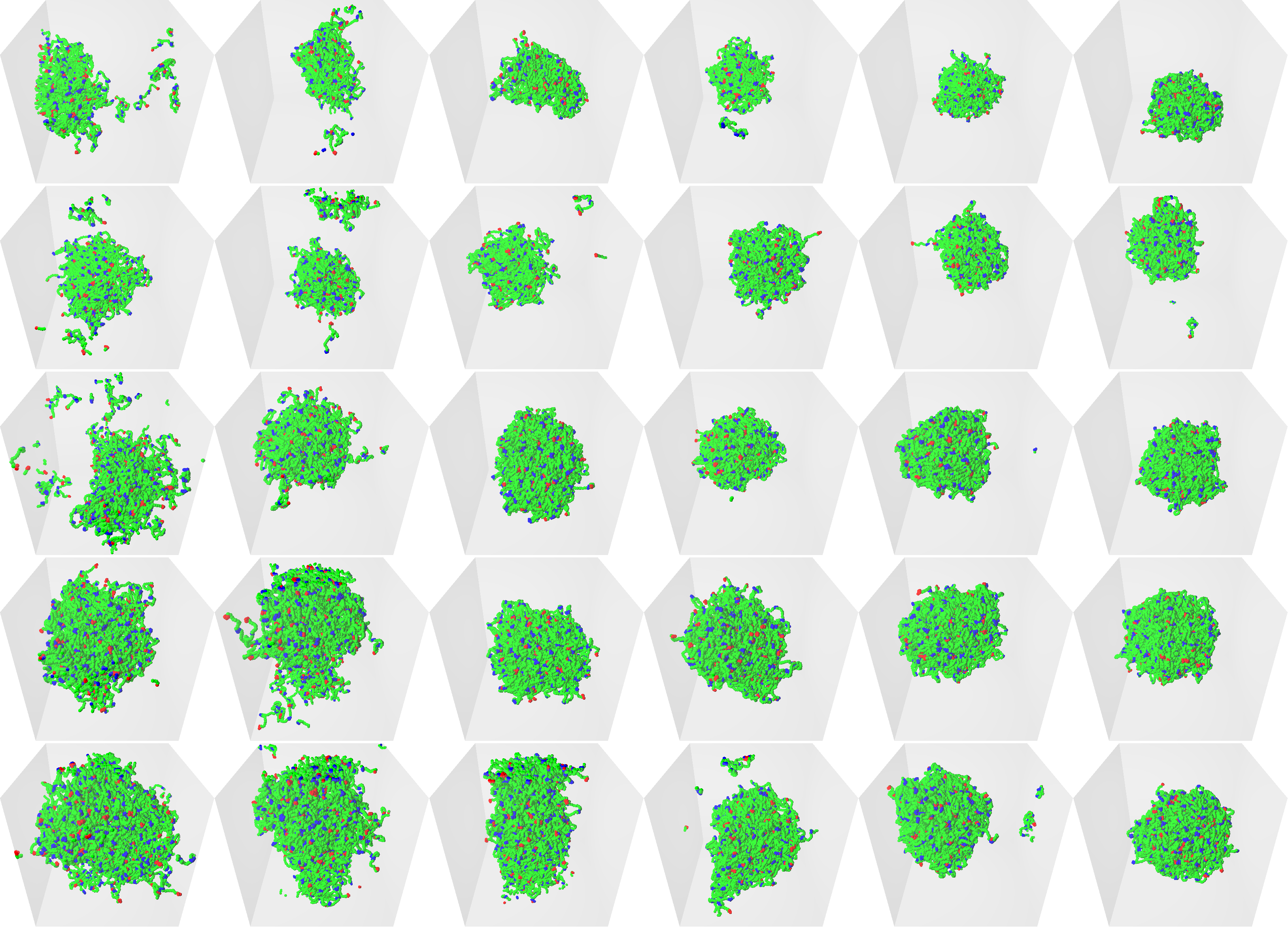

Supplement: Supplementary file 1 [file biology-12-00181-s001.zip › S2.png]

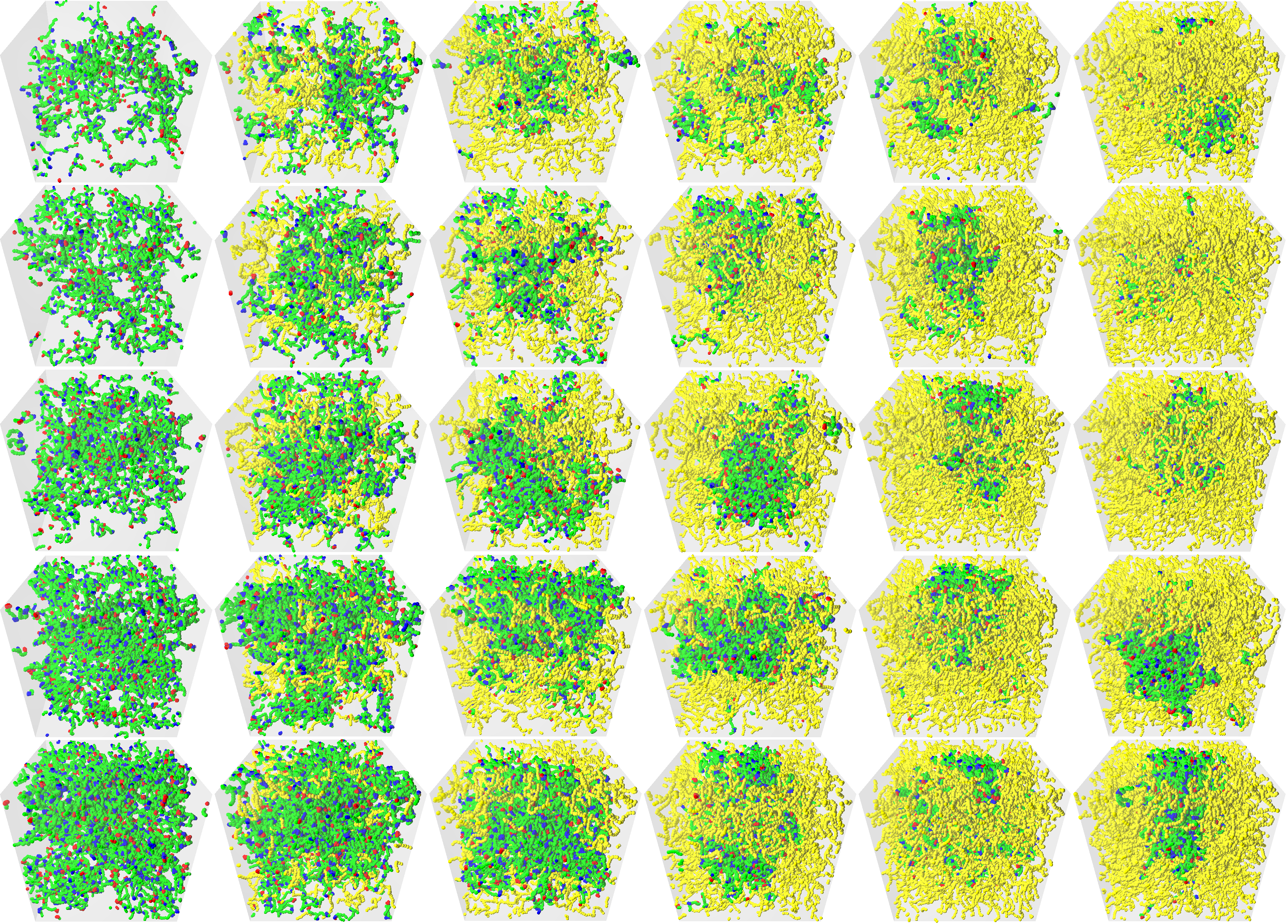

Supplement: Supplementary file 1 [file biology-12-00181-s001.zip › S3.png]
